# Determining the Ground Reaction Force Value and Location for Each Foot During Bipedal Stance Exercises from a Single Forceplate

**DOI:** 10.3390/s25154796

**Published:** 2025-08-04

**Authors:** Adrián Schmedling, Erik Macho, Francisco J. Campa, Ruben Valenzuela, Mikel Diez, Javier Corral, Paul Diego, Saioa Herrero, Charles Pinto

**Affiliations:** Bilbao School of Engineering, University of the Basque Country UPV/EHU, Plaza Ingeniero Torres Quevedo 1, 48013 Bilbao, Spain; erik.macho@ehu.eus (E.M.); fran.campa@ehu.eus (F.J.C.); ruben.valenzuela@ehu.eus (R.V.); mikel.diez@ehu.eus (M.D.); j.corral@ehu.eus (J.C.); paul.diego@ehu.eus (P.D.); saioa.herrero@ehu.eus (S.H.); charles.pinto@ehu.eus (C.P.)

**Keywords:** biomechanics, ground reaction force, center of pressure, bipedal stance, neural networks

## Abstract

In the study of biomechanical models, balance represents a complex problem due to the issue of indeterminate forces while standing. In order to solve this problem, it is essential to measure the ground reaction forces (GRFs) applied to each foot independently. The present work proposes a methodology for determining the independent GRF applied to each foot while standing when only one forceplate is available. For this purpose, an analytical method is proposed to determine the distribution of vertical GRFs and the position of the independent center of pressure (CoP) in each foot. Concurrently, several neural network (NN) models are trained to improve the results obtained. This hypothesis is experimentally validated by a self-developed device that allows one to simultaneously obtain the vertical GRF and CoP location of each foot at the same time that the GRF and the global CoP location are obtained from a single forceplate. The results obtained achieve a CoP position error of less than 8% and a vertical force error of 2%. The analytical hypothesis is demonstrated to offer a satisfactory level of precision, while the NN is shown to result in considerable improvement in some cases.

## 1. Introduction

One purpose of a biomechanical model is to understand the relationship between the desired motion and the forces that govern the body, including forces generated by the model actuators (muscles) and inertial forces that appear due to the movement and external forces applied, either disturbances or ground reaction forces (GRFs) and ground reaction moments [1]. These models can be used to assess different situations depending on the reading direction of Newton’s second axiom, which relates the force to the motion, through the inverse dynamics, which aims to study the reactions of a system under prescribed motion conditions, for example, by analyzing muscle activation during walking. They can also be used to assess these different situations through the direct dynamics in order to predict how the motion and the estimated forces will be affected by different changes in the model, including the effect of different surgeries or the application of orthoses [2].

One of the main tasks in locomotion is to maintain balance, which refers to the process of distributing weight uniformly. This enables an individual to maintain an upright and stable position, which allows them to maintain autonomy in their daily activities such as walking, running, or standing [3]. To achieve this, humans rely on the vestibular, visual, and somatosensory systems that provide the necessary information to maintain balance control processed by the central nervous system. These systems are responsible for producing the muscular activity necessary to develop the forces that allow the body to keep the center of mass within the area of the base of support [4].

Functional balance control is vital for the best possible quality of life. Cerebrovascular diseases, such as strokes, are a major pathological condition in contemporary society, representing one of the most significant causes of mortality, and they are responsible for the majority of disability-adjusted life years, representing a loss of 116.4 million years of quality of life in 2016 [5]. It is possible to evaluate a patient’s status through a variety of exercises, many of which are subjective in nature and depend on the interpretation of the evaluating physician. One method of obtaining a quantitative evaluation of the patient’s condition is to analyze their GRF during a series of exercises that have been predetermined [6].

A major challenge in the study and development of biomechanical models arises in cases of bipedal stance due to the establishment of a closed kinematic chain when both feet are in contact with the ground; in this scenario, the body’s skeletal system forms a redundantly actuated mechanism with more unknowns than equations, resulting in the joint forces and torques responsible for motion not being determinable [7]. This is particularly evident in the context of balance studies, where the subtlety of the movements performed during exercises can result in proportionally significant errors in the analysis of the results [8].

To be able to study a bipedal standing model, the reaction forces must be available for both feet independently, which means that it is necessary to know the force vector and the point of application on each foot separately. Numerous studies are dedicated to measuring or estimating the GRF in different circumstances, such as in environments outside the laboratory or those in which there is a lack of equipment due to economic limitations [9,10]. The main techniques employed can be separated into two categories, direct measurement with forceplates [11], treadmills [12], or insole pressure sensors [13], and estimation methods based on measurements from wearable sensors (IMUs) or videogrammetry [14,15], which make outdoor measurements possible and can be cheaper, although these lower costs result in reduced accuracy of the GRF obtained.

For the purpose of direct measurement of GRFs using a single forceplate, it is possible to obtain a single reaction, which is the sum of the action forces of both feet. However, discrimination between each foot’s ground reaction force is required to properly study biomechanical models using inverse dynamic analysis [16]. Currently, there are several articles proposing methods for obtaining the GRFs of each foot independently from the measurement of a single forceplate, but they all are applied in gait case studies, where most of the time (68% of the gait cycle), there is only one foot in contact with the ground (swing phase) while the other is in the air [17]. These studies do not adequately investigate static exercises in the bipedal stance [18], where both feet are in permanent contact with the ground.

In the research scope dedicated to predicting the GRFs, there is a notable presence of studies that are grounded on wearable IMU sensors that are attached to different body parts of the subject in order to record the orientation and velocity of each segment [19]. From this information, an inverse dynamic analysis of the model can be performed, resulting in an approximate measurement of the GRF [20]. The main issue with this methodology is that, in order to obtain results with a relevant degree of precision, it is necessary to analyze tests in which inertial forces have a significant effect. Consequently, it is only feasible for the study of gait or running; it is not a valid method for the assessment of equilibrium or quasi-static movements [19].

In contrast, research based on videogrammetry has the capability to accurately record body movements, especially when the subject is making slow movements, due to the high acquisition frequency of the equipment, over 1000 Hz [21]. However, this approach is limited by the excessive cost of the required cameras, in the case of marker systems, or by the inaccurate nature of markerless systems, an obstacle that significantly influences the accuracy of GRF estimation [22].

The analysis of biomechanical models is usually incredibly challenging due to the complexity of creating an accurate model and the fact that there are many variables involved in the problems under investigation. In cases where the correlation between the variables is simple, mechanical models are employed to perform computerized simulations to solve the equations. Alternatively, in cases where the correlation between variables is uncertain, the prevailing approach today is based on artificial intelligence (AI) models, which have demonstrated remarkable success in the last few decades [23].

In the field of artificial intelligence, a wide variety of models can be categorized depending on parameters such as their capacity (weak or strong), the modality of learning (supervised or unsupervised), and the type of algorithm employed (SVM, ANN, K-NN, etc.), among other criteria. These models have been extensively applied in the scientific field of Natural Language Processing (NLP), robotics, or biotechnology, among other domains, with neural networks (NN) being a prominent example due to their extensive functionality, including classification and regression methods [24].

Despite the amount of research that has been published about the use of AI as a tool for the study of biomechanics, around 80% of articles focus on classification tasks, while less than 12% focus on regression tasks [25]. Considering articles that use regression models, most studies use wearable sensors (IMUs and insole pressure sensors), motion capture systems, or electromyography sensors to assess the models. A frequent application is the prediction of the GRFs, or torques generated on a specific joint, derived from indirect measurements of these sensors [26]. Regarding the study of GRFs during quasi-static bipedal stance tests, such as those performed to assess balance, few articles are found, and none employ AI techniques to predict or assess the measured data.

This study forms part of the OREKA and BALANCE research projects of the University of the Basque Country (UPV/EHU), the primary aim of which is to evaluate the balance of patients afflicted with spasticity in the lower limbs after suffering a stroke. In line with this objective, two devices have been developed that are equipped with uniaxial sensor forceplates capable of measuring the patient’s center of pressure (CoP) as they perform a series of dynamic balance exercises in a bipedal stance [27].

In the majority of biomechanical modeling studies, it is essential to have the reaction forces of each foot as input data in terms of the value and position for each one. However, it is important to consider that there are devices that do not allow each foot to be measured independently, but only provide global values. In this paper, we propose novel methodologies for obtaining the individual data for each foot based on the global measurements and their validation, enabling low-budget device testing, as well as providing new methods for analyzing cases of bipedal standing exercises.

This paper is structured as follows: firstly, a prototype that has been built to measure both the global and individual GRF and CoP is described and validated. The next stage of the process involves the establishment of an analytical hypothesis to determine the individual CoPs from the global CoP, which is then evaluated through a set of experimental tests using the mentioned device. Finally, the results of these tests are used to train and compare multiple NN models, with the objective of obtaining more accurate results than those provided by the analytical model.

## 2. Materials and Methods

### 2.1. Analytical Hypothesis

To obtain the GRF for each independent foot, it is first necessary to determine the CoP locations of each one. To accomplish that using only one forceplate, it is necessary to make use of the subject’s overall CoP information. In this regard, [27] presents a simple method to deduce it from the resultant moment of the vertical forces measured in the four corners of the plate.

**Figure 1:**
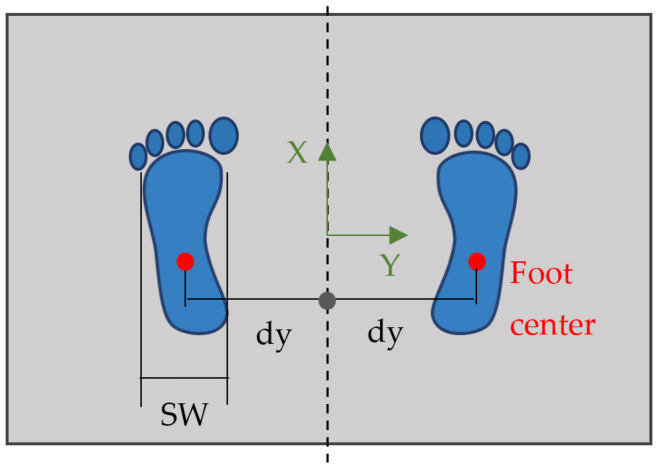
Feet position during the tests.

Once the global CoP has been calculated, it is necessary to assume certain conditions during the tests in order to be able to split it into two separate components: both feet must be placed in fixed, known positions, situated at equal distances (*dy*) from the X-axis of the platform to the midpoint of each sole, as shown in Figure 1, and the sole width (SW) must also be known. The Kistler coordinate system is used, since it is the most widely used in the field of biomechanical applications.

After the test parameters have been established, the following analytical hypothesis is proposed, where the anteroposterior displacement of each foot CoP is identical in both feet, and at the same time, they are equal to the anterior–posterior displacement of the global CoP (1).

(1) $${xCoP}^{global}={xCoP}^{left}={xCoP}^{right}$$

The mediolateral displacement of each foot’s CoP within the sole area is also considered to be identical for both feet and proportional to the ML displacement of the global CoP within the base of support (2) and (3), which is defined as the prescribed area between the subject’s two feet. It is necessary to shift the Y-axis coordinate of each foot by a distance *dy* so that the position of each foot is consistent with the global reference frame.

(2) $${yCoP}^{right}={yCoP}^{global}\cdot \frac{SW}{2dy+SW}+dy$$

(3) $$y{CoP}^{left}={yCoP}^{global}\cdot \frac{SW}{2dy+SW}-dy$$

To carry out this process correctly, the redistribution of forces between the two feet is fundamental. In this study, where the movements are quasi-static, horizontal forces are considered negligible, and only vertical forces are considered; therefore, a load factor (LF) (4) based on the proportional displacement of the CoP in the direction of the Y-axis relative to the width of the base of support is used for redistribution so that the result of the proposed method is consistent in terms of both positions and loads, according to expressions (5) and (6).

(4) $$LF=0.5+\frac{{yCoP}^{global}}{2dy+SW}$$

(5) $$F_{z}^{right}=F_{z}\cdot LF$$

(6) $$F_{z}^{left}=F_{z}\cdot \left(1-LF\right)$$

### 2.2. Measuring Device

#### 2.2.1. Description and Validation

**Figure 2:**
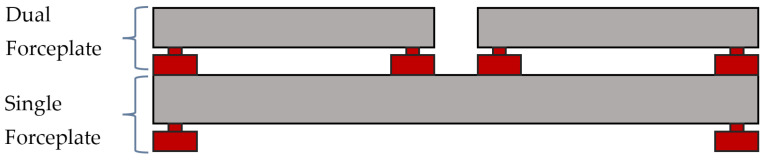
Schematic diagram of the device.

**Figure 3:**
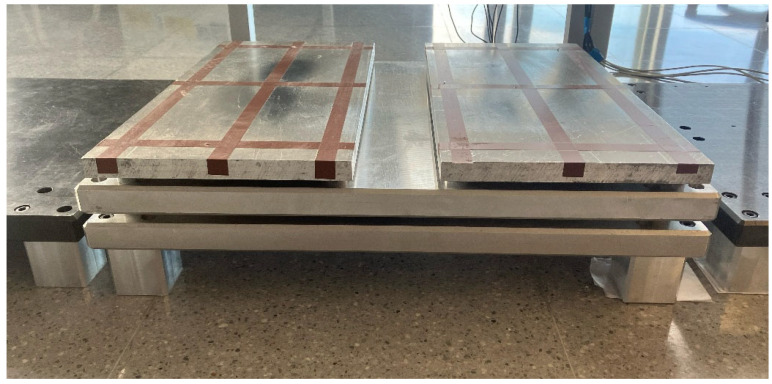
Double forceplate device.

To validate this analytical hypothesis, a double forceplate device has been developed that allows the simultaneous measurement of each individual foot’s CoP and ground reaction forces applied and the global CoP and reaction force. This methodology enables the comparison of the global CoP behavior with the individual CoP behavior of each foot during a test. The device comprises two forceplates: a single forceplate placed underneath and a dual forceplate located on top of the first one, as illustrated in Figure 2 and Figure 3.

The device architecture and hardware consist of an aluminum plate at the base, measuring 600 mm × 500 mm × 35 mm, containing four uniaxial INELTA sensors, model IMK-DMS-2410-2m-K, with a DMS-sensitivity of 1 ± 10% mV/V and a cut-off frequency of 1000 Hz, as well as two aluminum plates at the top, measuring 240 mm × 500 mm × 20 mm, one for each foot, each containing the same four sensors as the plate below. The signals are collected by a National Instruments compact DAQ model 9188, using NI 9239 analog cards (Emerson Electric Co., St. Louis, MO, USA), and processed through a computer with an Intel Core i5-8250U processor (Intel Technologies, Santa Clara, CA, USA) with 8 GB of RAM memory. MATLAB R2024b software is used with the data acquisition toolbox installed.

The sensors employed are uniaxial, meaning that only vertical reaction forces can be measured, and horizontal components must be assumed to be negligible. The plane where the GRF is applied is not the same plane where the sensors measure the reactions, resulting in a potential error in the CoP calculation when the applied force has a horizontal component. This error was estimated in Appendix A and was found to be negligible, since it does not exceed 1.7 mm.

**Figure 4:**
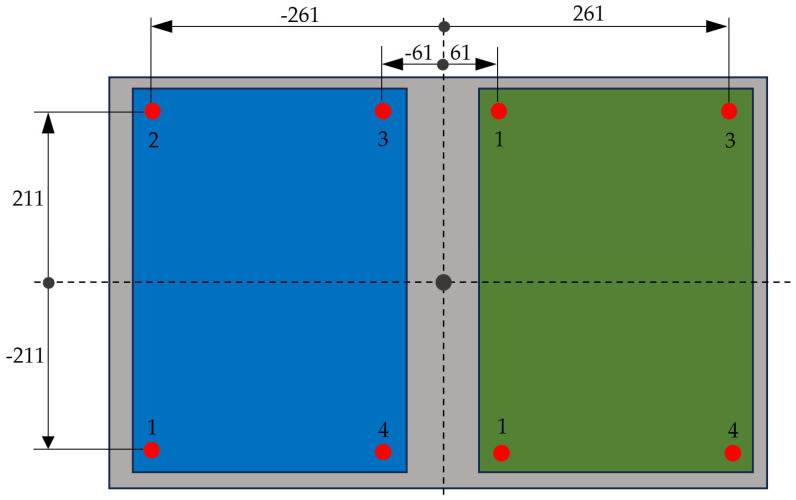
Scheme of sensor locations.

The sensors of the upper forceplates are in close proximity to the corners of each plate; their locations can be seen in Figure 4. The right plate sensors are located in symmetrical positions with respect to the Y-axis. In an analogous way, the sensors of the lower forceplate are located near the corners of the plate, directly below the four sensors at the outer corners of the two upper plates.

**Figure 5:**
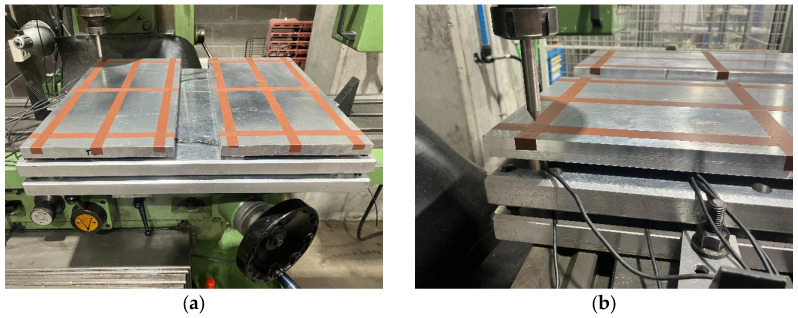
Device placed over the milling machine worktable (**a**) and with the tool (**b**).

**Figure 6:**
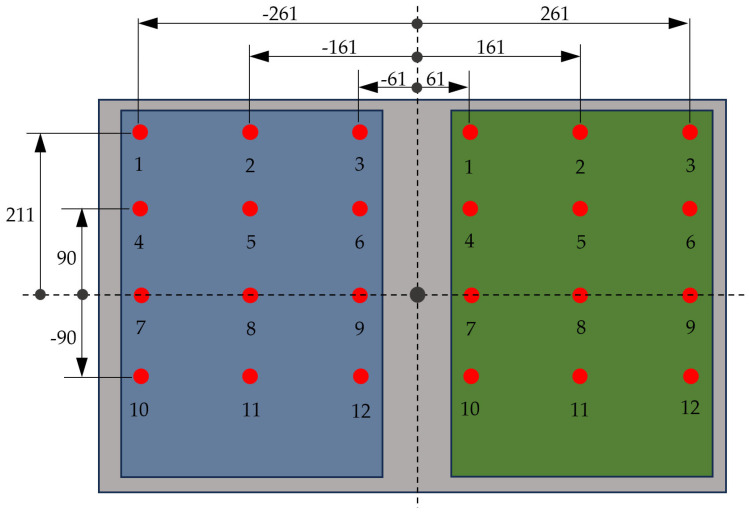
Scheme of points on which the load has been applied (units in mm).

To verify the accuracy and ensure the consistency of the results obtained with both plates, the device was placed on a milling machine worktable equipped with a special tool to apply force mounted to the chuck (Figure 5), allowing loads to be applied at precise locations with an error margin of ±0.01 mm. With this setup, a load between 200 N and 1000 N was applied at twelve different points on each of the two upper plates (see Figure 6), enabling the assessment of the error produced by each forceplate.

The tare process is not possible with the hardware employed; consequently, it is performed during signal processing. To ensure the accuracy of the tare, a protocol was followed in each test. Firstly, the device’s data are recorded for at least three seconds without applying any load. Then, the load is applied for another ten seconds. In this manner, the mean of the initial seconds of the recorded signal is subtracted, which results in the taring of the measurements. Subsequently, the data from these initial seconds until the load is applied are removed, leaving an almost constant signal from which the average location of the load on each plate is calculated.

The data were processed using a zero-phase Butterworth filter with a cut-off frequency located at 10 Hz. Given that the displacement of the CoP during the test of human body balance reacts to frequencies ranging between 0.5 and 1 Hz, the cut-off frequency is adequate to ensure noise suppression without losing any relevant information from the sensors [28].

**Figure 7:**
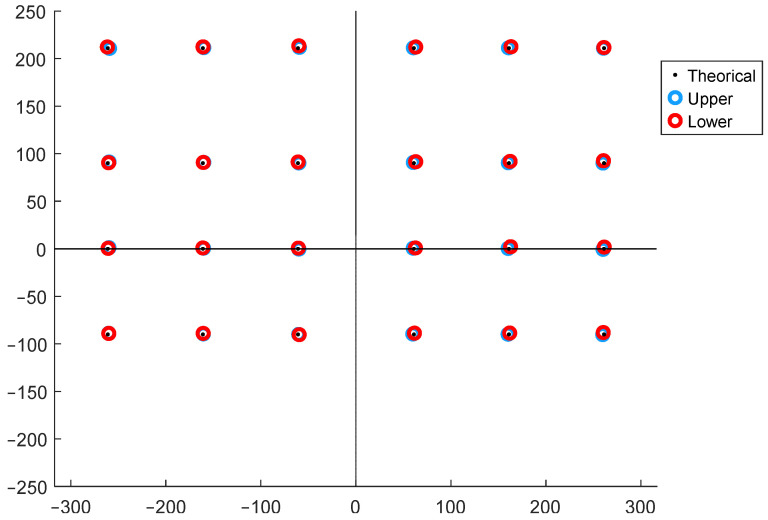
Graphical view of the validation test results (units in mm).

The outcomes of the experimental tests are presented in Figure 7, where the CoP location measured by the upper plate, the lower plate, and the actual points of application of the loads are shown in an overlay representation. The magnitude of the error committed is detailed in Table 1, where it is demonstrated that the mean error incurred by the left upper forceplate is 1.255 ± 0.42 mm, that incurred by the right upper forceplate is 0.934 ± 0.26 mm, and that incurred by the lower forceplate is 1.631 ± 0.69 mm, with a maximum value of 2.764 mm, which is marginally higher than the error incurred by the upper plates. This outcome proves the reliability and precision of the device developed.

#### 2.2.2. Experimental Protocol and Data Analysis

Once the device was validated, a series of tests were performed to assess the accuracy of the analytical hypothesis set out in Section 2.1. For this purpose, a group of ten healthy subjects—between 24 and 47 years old, 9 male and 1 female, with a weight of 76.76 ± 14.07 kg—performed a series of exercises described in Table 2.

These exercises have been designed to approximately reproduce the exercises that patients will perform on the OREKA machine platform (1, 2, and 3), as well as two control exercises (4 and 5) with the purpose of studying the most unfavorable cases for the analytical hypothesis in order to provide a solid framework for verification of the results obtained. Each test subject must repeat each test five times in order to obtain the relevant statistical data. In these exercises, the subject must perform displacements of their center of mass, which is a concentrated expression of the overall weight, while the CoP is the point where the plantar ground reaction force is applied, being displaced to keep the center of mass within the base of support [29].

To ensure repeatability and to maintain a valid CoP reference frame, users should always place their feet in the same position, at a width equal to twice the distance *dy*, and with their ankles aligned with the X-axis, as shown in Figure 1. The arms should hang naturally to the sides and should always hold an upright posture.

As long as the feet never slip or lift off the platform, which is a prerequisite for the reliable application of the analytical hypothesis, any necessary body movements can be performed during the exercise. It is also recommended that no sudden movements are made in order to minimize, as much as possible, the effects of inertial forces that could increase the measurement error.

Test data were collected and later processed using protocols similar to those described in Section 2.2.1 for the validation of the device, starting with the recording of a few seconds without any load to perform the tare. The user is then positioned and performs a squat before performing the exercise so that the starting time can be easily identified, ending with the recording of approximately 30 s of exercise movement. The data are processed by applying the Butterworth filter and then removing the time before the squat trigger of the acquisition in order to only keep the measured exercise data.

Once the test data are collected, they are treated by discarding the initial seconds of the acquisition, which are used to tare the measurement, and applying a low-pass filter at 10 Hz, as described in the previous section.

In order to verify the operation of the device under a real exercise condition, it is first assessed whether the global CoP measured by the lower plate matches that obtained from the measures by the upper plates during the tests according to expression (7).

(7) $$x,yCoP=x,y{CoP}^{left}\cdot \frac{F_{z}^{left}}{F_{z}}+{x,yCoP}^{right}\cdot \frac{F_{z}^{right}}{F_{z}}$$

To assess the performance of the equations, three indicators are used: the root mean square error (RMSE) (8), which is an overall error magnitude; the normalized RMSE (NRMSE) (9), which evaluates the relative error with respect to the actual range of variation and is a more representative indicator of the system’s accuracy; and the Pearson correlation coefficient (10), which determines the linear correlation between two sets data:

(8) $$RMSE=\sqrt{\frac{1}{n}\sum_{i=1}^{n}{\left(x_{i}^{R}-x_{i}^{P}\right)}^{2}}$$

(9) $$NRMSE=\frac{RMSE}{ref}\cdot 100$$

(10) $$COR=\frac{\sum_{i=1}^{n}\left(x_{i}^{R}-{\bar{x}}^{R}\right)\left(x_{i}^{P}-{\bar{x}}^{P}\right)}{\sqrt{\sum_{i=1}^{n}{\left(x_{i}^{R}-{\bar{x}}^{R}\right)}^{2}\sum_{i=1}^{n}{\left(x_{i}^{P}-{\bar{x}}^{P}\right)}^{2}}}$$

where $x^{R}$ refers to the real measured signal values, $x^{P}$ refers to the predicted signal values, n is the number of time steps, and $\bar{x}$ is the mean value of the signal for a specific test. ref is a reference of the maximum value of the variable for each test subject: the foot length for the xCoP, the foot width for the yCoP, and the weight for the Fz.

As can be seen in Table 3, the mean values of the RMSE between the upper and lower plates measurements are 0.655 ± 0.22 mm for the xCoP, 0.898 ± 0.49 mm for the yCoP, and 1.72 ± 0.5 N for the vertical reaction force.

From this point onwards, all tables and figures express RMSE and CoP measurements in millimeters (mm), force measurements in Newtons (N), NRMSE in percentage (%), and the COR coefficient as a dimensionless value ranging between −1 and 1.

To normalize the error, the xCoP data are divided by each subject’s foot length; the yCoP data are divided by the foot width (ball width), measured as described in [30]; and the vertical reaction forces are divided by the subject’s weight, providing the NRMSE.

The results presented in Table 4 indicate that the relative error of the xCoP is less than 0.4% in any case, as for the Fz measurement, which represents a very accurate result. In the case of yCoP displacement, the error is slightly higher but remains below 4%, which again confirms the high accuracy of the device verified in Section 2.2.1., even in cases where moving loads are applied simultaneously to both upper plates.

In all instances, the correlation coefficient shown in Table 5 for all variables exceeds 0.98, with the exception of the vertical reaction force in the ML test, where it drops to 0.927, a result that remains highly accurate, again demonstrating the satisfactory performance of the device.

**Figure 8:**
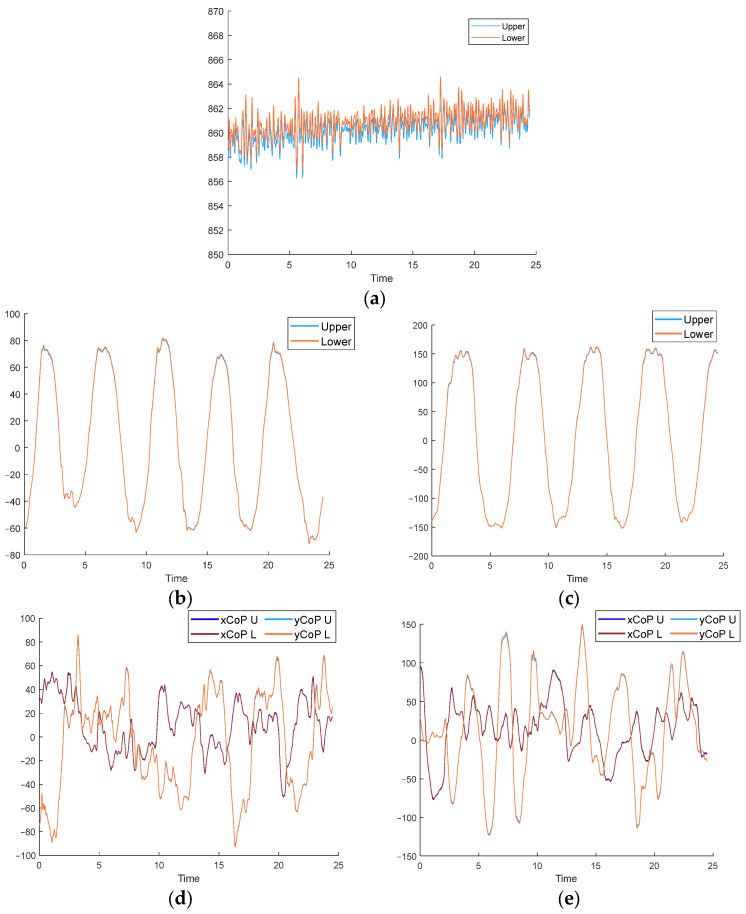
Device validation with exercises of one test subject. (**a**) Fz on static test. (**b**) xCoP on AP test. (**c**) yCoP on ML test. (**d**) xCoP and yCoP on rotation test. (**e**) xCoP and yCoP on random test.

After evaluating the correct operation of the device, as can be seen in Figure 8, the accuracy of the analytical expressions proposed for each variable was assessed according to the three previously mentioned indicators in each type of test exercise.

As shown in Table 6, Table 7 and Table 8, both the NRMSE and the correlation are better when calculating the xCoP than the yCoP, except in the rotation exercises, where the error in the xCoP increases significantly. This exception is consistent with the nature of the analytical model’s hypothesis, which assumes that the displacement of the CoP in the AP direction of both feet must be equal to the displacement of the global CoP; in the rotation exercise, where the sum of the displacements of both feet must result in a displacement that is nearly zero, the model is incapable of predicting the actual displacement of each foot separately.

In the remaining exercises, the outcome is consistent, as the physiognomy of the feet, which are approximately 2.5 times longer than they are wide, means that minor errors in the mediolateral direction have a proportionally greater effect than those made in the anteroposterior direction. Furthermore, due to the anatomical characteristics of the human body, maintaining equilibrium in the ML direction is more challenging [31], so the yCoP exhibits more erratic and harder-to-model movements, which is reflected in the low correlation between the measured yCoP signal of each foot and the signal predicted from the global yCoP.

In consideration of the findings of [32], it is evident that accurate measurement of the GRFs is crucial in the construction of biomechanical models, since small variations in the torque generated at the ankle can lead to large deviations in the torque generated at the knee and hip. Therefore, although the model may be valid for use in simple exercises, the error is too large to be applied to a general case.

For the exercises programmed on the OREKA machine (STC, AP, and ML), the NRMSE of the yCoP exceeds 6%, with a poor correlation coefficient. In contrast, the xCoP demonstrates a substantially reduced error rate, although the correlation is unsatisfactory except for the AP exercise, where a value greater than 0.9 is obtained, which is regarded as an acceptable value. The Fz of each foot has the lowest NRSME, under 2%, and the highest correlation for these exercises. This is of particular significance given that the value of this variable is critical for the correct calculation of the GRF.

Considering the results obtained, it can be concluded that, although the analytical hypothesis can adequately model the distribution of vertical GRFs between the two feet, it does not have the same level of accuracy in modeling the local CoP displacement, especially for exercises that do not fully meet the hypothesis assumptions (ROT and RND). As a result, the hypothesis is only acceptable for determining the Fz and the xCoP in AP exercises.

### 2.3. Neural Network Development

As the purpose of this work is to be applied in biomechanical models, where the measurement of the GRFs has a significant impact on the outcome analysis, to obtain better results than those offered by the analytical hypothesis, especially in the general cases where its performance is very poor, the device developed and the data from the tests carried out were used to train a neural network (NN) capable of predicting the distribution of GRFs in both feet using only the global CoP and vertical reaction force data.

Given the lack of an established methodology for determining the optimal NN configuration for this specific application, different NN models were trained, varying the number of layers, the number of neurons per layer, and the number of epochs, as shown in Table 9. The models were trained using four different datasets, each consisting of four out of the five repetitions of the ten test subjects, while the remaining test repetition was used to validate the models:

- Dataset 1: 1 exercise (AP) × 4 repetitions × 10 subjects.
- Dataset 2: 1 exercise (ML) × 4 repetitions × 10 subjects.
- Dataset 3: 3 exercises (STC/AP/ML) × 4 repetitions × 10 subjects.
- Dataset 4: 5 exercises (STC/AP/ML/ROT/RND) × 4 repetitions × 10 subjects.

**Table 9:** Neural network model parameters (- means that the layer does not exist).

Network Number	Layer 1 Neurons	Layer 2 Neurons	Layer 3 Neurons	Number of Epochs
1	10	-	-	750
2	50	-	-	750
3	100	-	-	500
4	10	10	-	750
5	10	25	-	750
6	10	50	-	750
7	25	50	-	500
8	10	10	10	750
9	10	10	25	500
10	10	25	50	250

This methodological approach is used to assess whether the data generated by a specific NN for the main exercises is better than that of a general-purpose NN. Also, it allows us to evaluate whether the control tests (ROT and RND) of the analytical hypothesis can be used to improve the performance of the network, or whether they lead to overfitting, introducing further error to the model output.

The NNs were created using MATLAB software with the Deep Learning Toolbox. The remaining hyperparameters used in this study were defined by default in the “fitnet” function:

- Train function: Levenberg–Marquardt.
- Transfer function: tangent sigmoid.
- Loss function: MSE.
- Dataset division (train/validation/test): 70%/15%/15%, randomly chosen.

## 3. Results

**Figure 9:**
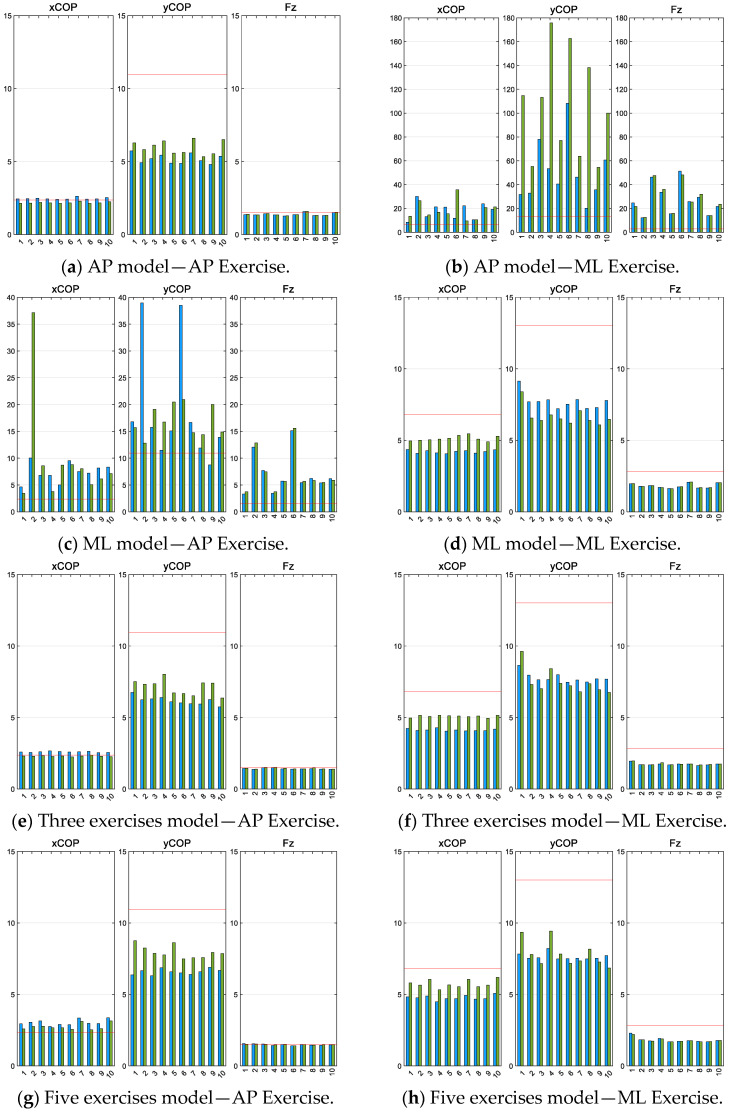
Comparison of NRMSE for the AP and ML exercises of the model trained with the AP dataset (**a**,**b**), with the ML dataset (**c**,**d**), with the three basic datasets (static, Ap, and ML) (**e**,**f**), and with the five datasets (**g**,**h**). The red line represents the mean outcome from the analytical expression. The blue bars represent the results of the left foot for each NN model, while the green bars represent the right foot.

After training all the models with the different datasets presented in the previous chapter, their performance was evaluated in the same way as the analytical model was evaluated in Section 2.2.2. Initially, the accuracy of the specific models for the AP and ML exercises was assessed in comparison to the accuracy of the general models for their respective exercises. This was performed to determine whether training a specific NN results in a significant enhancement in the outcomes or not. The results are shown in Figure 9.

The findings indicate that the NN models trained specifically for each exercise provide satisfactory results when evaluating the exercise they were trained on. However, they are unable to achieve the same level of performance when evaluating an exercise they were not trained for. In contrast, the models that were trained for general purposes demonstrate a high level of accuracy for both exercises that is comparable to the accuracy of the models specifically developed for this purpose. Consequently, it can be concluded that the specific models do not offer any advantage over the general models, apart from being faster to train due to requiring fewer datasets. This is because they are only accurate for one specific exercise without offering greater precision than the general-purpose models.

In general, models that have been trained with all five types of tests appear to achieve a slightly higher level of performance when compared with models that have been trained exclusively with the three main datasets. However, when only the main exercises are taken into consideration, the three exercises dataset model produces more favorable results. Moreover, there are no significant disparities between the various models trained with the same dataset. Based on the results obtained, the subsequent analysis focuses exclusively on the NNs trained with only three datasets, for which the results of each model are presented in Table 10, Table 11, Table 12 and Table 13.

After determining the optimal dataset for training, a detailed analysis of the output data from the NNs with respect to their hyperparameters was conducted. This analysis yielded the results shown in Table 14, Table 15, Table 16, Table 17 and Table 18 for each of the five exercises performed.

**Figure 10:**
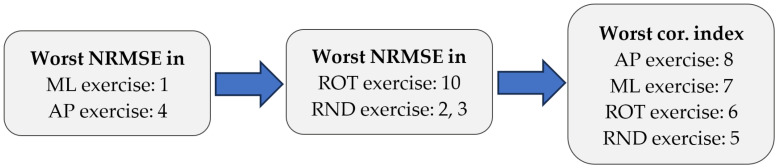
Flowchart of the final model selection process.

Given the similar outcomes obtained by all models, the selection of the most appropriate one was based on the elimination of those that yielded the worst results, as shown in Figure 10. First, the models that produced the highest NRMSE in the AP and ML exercises were eliminated, since these are the exercises performed by the OREKA machine. According to this criterion, models 1 and 4 were initially excluded. Secondly, the models that produced the highest NRMSE for the remaining exercises were discarded. These are models 2, 3, and 10. To finalize the selection, the models with the worst correlation index, 5, 6, 7, and 8, were eliminated. This analysis indicates that the optimal model is number 9, as indicated in Table 9, corresponding to a two-layer model, one with 10 neurons and one with 25.

The most significant results obtained from this analysis process, corresponding to NN model 9, are summarized in Table 19 and Table 20, where they are compared with the results obtained through the analytical hypothesis.

## 4. Discussion

**Figure 11:**
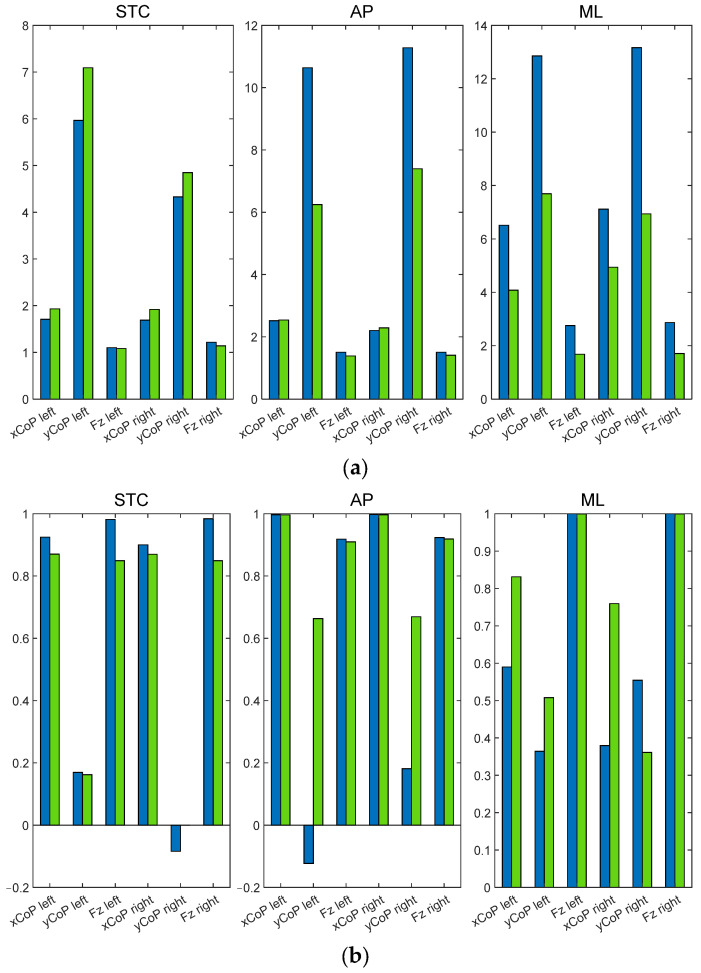
Graphic comparison of the analytical (blue bars) and NN (green bars) results. (**a**) NRMSE in percentage. (**b**) Correlation index.

Once the optimal training method is established and the most effective NN is proposed, the next stage is to evaluate the results provided by the NN method against those obtained using the analytical hypothesis and considering the real measurements collected during the validation exercises. This comparison is shown in Table 19 and Table 20 and in Figure 11.

A general conclusion that can be drawn from the results of this study is that it is possible to obtain both the vertical GRF and the CoP position of each foot independently from a single dynamometric platform during a bipedal stance exercise.

The error observed is highly dependent on the variable of interest, with the lateral displacement of the CoP being the most difficult to determine due to the relatively small size of the foot in that direction, thereby causing small errors to be proportionally large. In contrast, it was demonstrated that the proposed analytical hypothesis is valid for characterizing both the displacement of the xCoP and the Fz that falls on each foot in AP exercises. This situation corresponds with 2D biomechanical models with the sagittal plane as the plane of symmetry, which is a common case of study.

With regard to the assessment of the yCoP, the analytical hypothesis has proven unable to achieve an error margin below 10% across the analyzed exercises, except for the static exercise. The use of NN has been shown to improve the accuracy of the results, particularly in the case of yCoP evaluation and during ML exercises. This approach has been observed to reduce the error by nearly half, significantly improving the correlation between the predicted and measured values.

**Figure 12:**
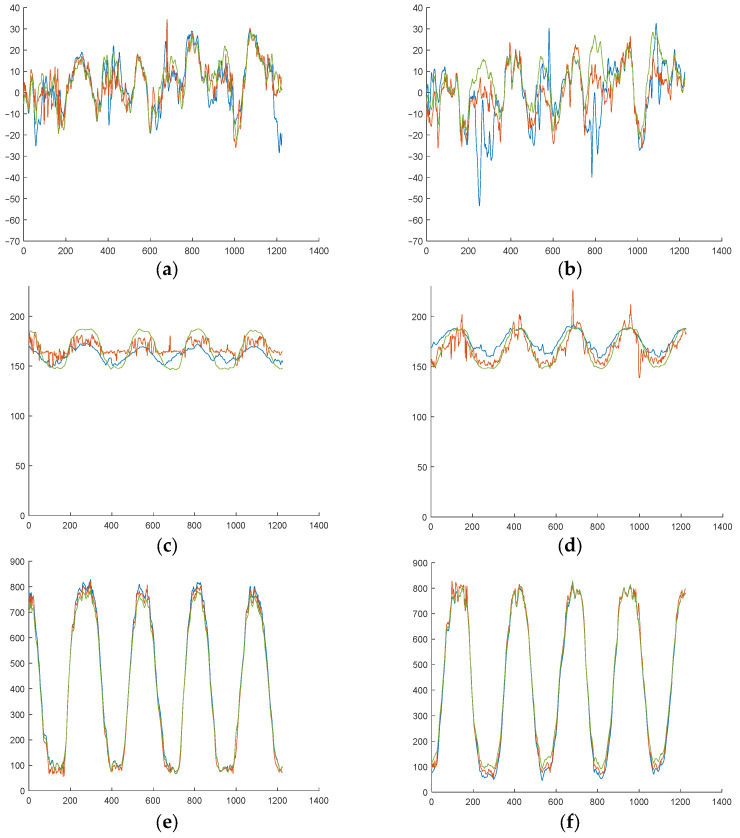
Subject 1 results during an ML exercise. (**a**) xCoP left. (**b**) xCoP right. (**c**) −yCoP left. (**d**) yCoP right. (**e**) Fz left. (**f**) Fz right. Real measure is shown in blue, that predicted analytically is shown in green, and that predicted by the NN is shown in red.

**Figure 13:**
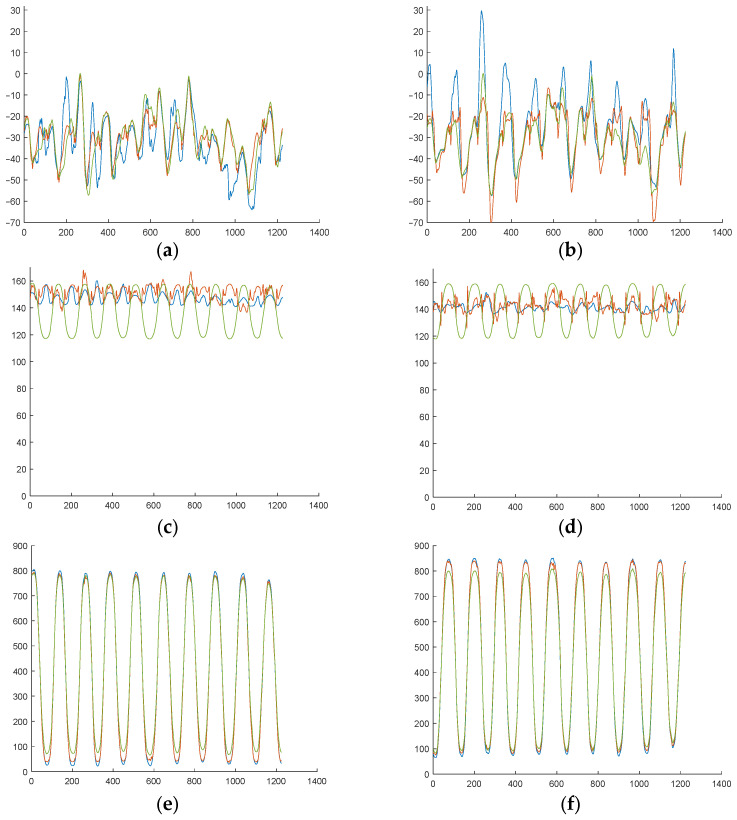
Subject 7 results during an ML exercise. (**a**) xCoP left. (**b**) xCoP right. (**c**) −yCoP left. (**d**) yCoP right. (**e**) Fz left. (**f**) Fz right. Real measure is shown in blue, that predicted analytically is shown in green, and that predicted by the NN is shown in red.

It is necessary to remark that these results represent aggregate means derived from the various tests conducted on the ten volunteer subjects. However, when the results are analyzed on a more individualized approach, it is possible to observe cases where the analytical model exhibits a high degree of correlation with the measurements and where the NN does not provide a substantial improvement (Figure 12c,d). Conversely, in other trials, the analytical hypothesis does not yield such favorable results, and in these cases, the NN models provide a substantial improvement (see Figure 13c,d).

**Figure 14:**
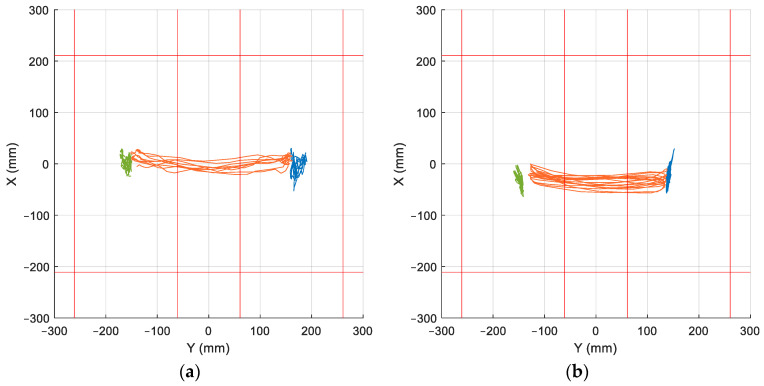
Statokinesigram of two subjects representing the global CoP (orange) and the left and right individual CoP of each foot (green and blue) during an ML exercise. (**a**) Subject 1. (**b**) Subject 7.

This finding proves that individuals may employ diverse strategies when performing the same exercise. As illustrated in Figure 14b, the subject’s statokinesigram during an ML exercise reveals the global CoP moving laterally while the CoP of each independent foot moves in the AP direction, thereby contradicting the analytical hypothesis. In contrast, Figure 14a presents another subject performing the same exercise, demonstrating better alignment with the analytical hypothesis, as evidenced by the movement of the individual CoP of each foot in both directions.

## 5. Conclusions

In conclusion, this study demonstrates the feasibility of determining the GRFs applied to each independent foot during standing tests using a single forceplate, which are challenging to solve due to the indeterminacy problem, particularly in circumstances where resources are scarce.

As would be expected, the displacement of the global CoP and that of the individual CoP of each foot has a strongly linear correlation. This explains why all NN models yield similar results, regardless of the number of layers and neurons, and it is more significant to select a dataset appropriate to the task.

Consequently, in studies using 2D models in the sagittal plane where AP exercises are performed, the analytical hypothesis can be reasonably accurate in solving the problem. However, in general cases, it has been observed that the result is highly dependent on the subject’s behavior during the test. In such instances, NNs have proven to be a more effective tool for estimating forces, particularly with regard to the displacement of the yCoP, thereby significantly improving upon the analytical approach.

The findings emphasize the necessity of taking into consideration individual variations in the postural control response when performing certain exercises. It is imperative to understand how users adapt their postural control strategies during the exercise, as this may yield valuable information for optimizing predicted results, thereby facilitating the development of more effective models and more accurate simulations of biomechanical problems.

Future research will focus on performing these same trials with hemiplegic patients to evaluate the results obtained from the analytical hypothesis and the NN model. If it is found that the error is significantly larger in these subjects when performing the test, it would be necessary to retrain the NN and re-evaluate the results. This would require the development of a new specialized model to evaluate subjects with specific physical features.

Moreover, the findings of this study can be employed as a practical application of the measurements obtained from tests carried out on the OREKA machine, detailed in articles [3,27], which is currently being evaluated in clinical trials. This device is equipped with a unique platform designed to assess the balance of stroke patients suffering from hemiplegia. The results of this work will be used to obtain the GRF of each individual foot so that this information can then be processed using biomechanical analysis software, such as OpenSim, to determine the lower body muscle activation of patients during the test exercises.

## Figures and Tables

**Table 1:** Validation test results.

	Error (mm)					
**UPPER** **FORCEPLATES**	1.724	1.091	1.728	0.631	0.558	0.549
	1.879	1.287	0.979	1.015	0.86	1.072
	1.627	0.865	1.014	0.974	0.92	1.103
	1.456	0.672	0.735	1.161	0.994	1.416
**LOWER** **FORCEPLATE**	1.328	1.331	2.609	2.289	2.629	0.641
	0.97	0.892	1.273	2.408	2.303	2.641
	0.624	0.878	0.896	1.819	2.764	1.824
	1.381	1.11	1.397	1.411	1.607	2.112

**Table 2:** Test exercise descriptions.

Exercise Number	Movement	Description
*1*	Static (STC)	Maintain a relaxed pose without movement
*2*	Antero-Posterior (AP)	Displace the center of mass forward and backward, distributing the load equally between the two feet
*3*	Medio-Lateral (ML)	Displace the center of mass side to side, distributing the load alternately between the two feet
*4*	Rotation (RTN)	Rotation of the trunk, distributing the load alternately between one foot toe and other foot heel
*5*	Random (RND)	Random movement of the body without lifting the feet

**Table 3:** RMSE between upper forceplate and lower forceplate.

	xCoP	yCoP	Fz
**STC**	0.385	0.344	1.036
**AP**	0.915	0.485	1.381
**ML**	0.567	1.447	2.207
**ROT**	0.548	0.879	1.85
**RND**	0.860	1.333	2.125

**Table 4:** NRMSE between upper forceplate and lower forceplate.

	xCoP	yCoP	Fz
**STC**	0.149	0.33	0.136
**AP**	0.359	0.465	0.18
**ML**	0.221	1.384	0.281
**ROT**	0.214	0.835	0.237
**RND**	0.335	1.283	0.272

**Table 5:** Correlation coefficients between upper forceplate and lower forceplate.

	xCoP	yCoP	Fz
**STC**	1.000	1.000	0.998
**AP**	1.000	0.999	0.981
**ML**	0.999	1.000	0.927
**ROT**	1.000	1.000	0.993
**RND**	1.000	1.000	0.993

**Table 6:** RMSE of the analytical expression.

	xCoP Left	yCoP Left	Fz Left	xCoP Right	yCoP Right	Fz Right
**STC**	4.458	6.302	8.116	4.404	4.493	8.998
**AP**	6.485	11.19	11.55	5.670	11.89	11.66
**ML**	16.73	13.48	20.22	18.37	13.87	21.07
**ROT**	51.26	12.59	23.03	49.58	14.66	24.05
**RND**	17.58	13.59	18.82	17.10	11.03	19.23

**Table 7:** NRMSE of the analytical expression in percentage.

	xCoP Left	yCoP Left	Fz Left	xCoP Right	yCoP Right	Fz Right
**STC**	1.708	5.968	1.103	1.690	4.330	1.219
**AP**	2.520	10.63	1.504	2.203	11.28	1.505
**ML**	6.512	12.85	2.758	7.114	13.16	2.867
**ROT**	19.91	11.92	2.968	19.28	13.89	3.095
**RND**	6.840	12.95	2.502	6.654	10.50	2.552

**Table 8:** Correlation coefficients of the analytical expression.

	xCoP Left	yCoP Left	Fz Left	xCoP Right	yCoP Right	Fz Right
**STC**	0.924	0.170	0.982	0.900	−0.080	0.984
**AP**	0.996	−0.121	0.919	0.997	0.181	0.924
**ML**	0.590	0.364	1.000	0.380	0.555	1.000
**ROT**	0.131	−0.052	0.914	0.409	0.156	0.910
**RND**	0.822	0.340	0.997	0.856	0.428	0.997

**Table 10:** Mean results for each variable and each indicator for AP dataset-trained NN.

	Model	1	2	3	4	5	6	7	8	9	10
**RMSE**	**xCoP**	22.4	38.0	27.1	35.7	29.9	34.6	27.3	22.1	33.3	32.1
	**yCoP**	33.6	22.8	44.2	53.9	28.8	56.1	27.8	37.1	25.0	39.1
	**Fz**	76.9	44.6	144	127	55.8	141	83.1	94.8	56.5	82.3
**NRMSE**	**xCoP**	8.67	14.7	10.5	13.8	11.6	13.4	10.6	8.57	12.9	12.4
	**yCoP**	32.1	21.7	42.0	51.5	27.5	53.2	26.4	35.3	23.9	37.3
	**Fz**	9.99	6.02	19.1	15.8	7.21	18.7	10.8	12.5	6.91	11.1
**COR**	**xCoP**	0.62	0.46	0.57	0.61	0.53	0.56	0.51	0.64	0.53	0.54
	**yCoP**	0.30	0.15	0.33	0.19	0.28	0.34	0.32	0.42	0.18	0.16
	**Fz**	0.84	0.89	0.46	0.92	0.84	0.36	0.74	0.76	0.91	0.74

**Table 11:** Mean results for each variable and each indicator for ML dataset-trained NN.

	Model	1	2	3	4	5	6	7	8	9	10
**RMSE**	**xCoP**	19.3	31.3	23.0	22.4	22.5	23.8	22.8	21.9	23.0	23.1
	**yCoP**	12.9	15.2	13.4	12.9	13.1	15.5	12.9	11.3	12.6	12.0
	**Fz**	24.1	36.1	31.9	28.1	26.0	44.7	31.3	31.7	32.7	27.9
**NRMSE**	**xCoP**	7.50	12.3	8.96	8.69	8.73	9.21	8.87	8.54	8.99	9.01
	**yCoP**	12.4	14.7	12.8	12.3	12.5	14.9	12.4	10.9	12.1	11.5
	**Fz**	3.12	5.32	4.22	3.53	3.35	5.80	4.25	4.19	4.45	3.74
**COR**	**xCoP**	0.77	0.67	0.69	0.74	0.70	0.68	0.64	0.74	0.70	0.66
	**yCoP**	0.46	0.30	0.24	0.26	0.29	0.36	0.20	0.26	0.34	0.22
	**Fz**	0.88	0.79	0.74	0.84	0.82	0.81	0.75	0.81	0.81	0.74

**Table 12:** Mean results for each variable and each indicator for 3-dataset-trained NN.

	Model	1	2	3	4	5	6	7	8	9	10
**RMSE**	**xCoP**	18.3	20.2	19.6	18.7	18.7	18.9	18.7	19.7	18.6	19.8
	**yCoP**	10.5	10.9	10.9	10.3	9.74	9.65	9.97	9.62	9.62	10.1
	**Fz**	18.1	20.9	21.7	19.1	18.3	17.2	19.0	16.7	16.3	18.2
**NRMSE**	**xCoP**	7.09	7.82	7.63	7.28	7.25	7.35	7.26	7.67	7.21	7.69
	**yCoP**	10.0	10.5	10.5	9.89	9.32	9.23	9.53	9.22	9.19	9.65
	**Fz**	2.35	2.68	2.88	2.6	2.37	2.32	2.48	2.27	2.13	2.36
**COR**	**xCoP**	0.76	0.74	0.73	0.77	0.75	0.75	0.73	0.75	0.75	0.70
	**yCoP**	0.43	0.27	0.29	0.34	0.29	0.34	0.36	0.29	0.32	0.33
	**Fz**	0.95	0.92	0.89	0.94	0.93	0.92	0.91	0.93	0.93	0.90

**Table 13:** Mean results for each variable and each indicator for 5-dataset-trained NN.

	Model	1	2	3	4	5	6	7	8	9	10
**RMSE**	**xCoP**	18.5	18.7	19.1	18.4	18.5	18.3	19.7	18.2	18.5	19.9
	**yCoP**	9.28	8.77	8.54	9.14	8.75	8.68	8.66	8.71	8.75	8.90
	**Fz**	14.8	14.0	13.9	14.3	13.8	13.9	14.0	13.9	13.7	14.2
**NRMSE**	**xCoP**	7.18	7.26	7.41	7.15	7.16	7.11	7.63	7.07	7.16	7.73
	**yCoP**	8.89	8.38	8.15	8.74	8.36	8.29	8.27	8.32	8.35	8.50
	**Fz**	1.93	1.82	1.82	1.86	1.81	1.81	1.84	1.81	1.79	1.85
**COR**	**xCoP**	0.77	0.76	0.71	0.78	0.77	0.75	0.67	0.78	0.76	0.68
	**yCoP**	0.48	0.43	0.37	0.47	0.41	0.35	0.36	0.44	0.33	0.39
	**Fz**	0.96	0.94	0.92	0.96	0.95	0.93	0.92	0.96	0.94	0.92

**Table 14:** Mean results for three-dataset-trained NN in static exercise.

	Model	1	2	3	4	5	6	7	8	9	10
**NRMSE**	**xCoP**	1.79	1.58	1.74	1.65	1.67	1.88	1.85	1.97	1.93	1.93
	**yCoP**	7.53	5.74	5.91	6.24	6.30	6.79	6.28	5.91	5.97	6.28
	**Fz**	1.15	1.03	1.19	1.21	1.17	1.30	1.38	1.06	1.11	1.28
**COR**	**xCoP**	0.91	0.89	0.89	0.92	0.90	0.87	0.78	0.92	0.87	0.79
	**yCoP**	0.60	−0.11	−0.10	0.31	−0.04	0.13	0.21	0.01	0.08	0.03
	**Fz**	0.95	0.84	0.71	0.95	0.86	0.81	0.79	0.89	0.85	0.70

**Table 15:** Mean results for three-dataset-trained NN in AP exercise.

	Model	1	2	3	4	5	6	7	8	9	10
**NRMSE**	**xCoP**	2.44	2.42	2.46	2.47	2.47	2.42	2.46	2.50	2.41	2.41
	**yCoP**	7.13	6.78	6.83	7.21	6.41	6.35	6.24	6.68	6.82	6.05
	**Fz**	1.45	1.39	1.50	1.50	1.43	1.40	1.40	1.45	1.40	1.38
**COR**	**xCoP**	1.00	1.00	1.00	1.00	1.00	1.00	1.00	1.00	1.00	1.00
	**yCoP**	0.74	0.69	0.66	0.66	0.71	0.71	0.70	0.63	0.67	0.70
	**Fz**	0.93	0.92	0.90	0.89	0.91	0.91	0.90	0.89	0.91	0.91

**Table 16:** Mean results for three-dataset-trained NN in ML exercise.

	Model	1	2	3	4	5	6	7	8	9	10
**NRMSE**	**xCoP**	4.60	4.62	4.60	4.71	4.58	4.62	4.56	4.59	4.51	4.67
	**yCoP**	9.13	7.64	7.33	8.04	7.70	7.34	7.22	7.41	7.32	7.22
	**Fz**	1.96	1.70	1.70	1.79	1.69	1.74	1.75	1.66	1.70	1.75
**COR**	**xCoP**	0.77	0.78	0.79	0.79	0.80	0.79	0.79	0.78	0.80	0.78
	**yCoP**	0.33	0.42	0.45	0.37	0.38	0.46	0.48	0.46	0.43	0.49
	**Fz**	1.00	1.00	1.00	1.00	1.00	1.00	1.00	1.00	1.00	1.00

**Table 17:** Mean results for three-dataset-trained NN in rotation exercise.

	Model	1	2	3	4	5	6	7	8	9	10
**NRMSE**	**xCoP**	19.1	19.8	19.9	18.9	19.5	19.5	19.6	19.3	19.3	20.3
	**yCoP**	11.4	13.3	12.8	12.1	12.8	12.7	13.1	12.7	13.3	13.6
	**Fz**	2.94	3.04	3.13	2.98	3.28	3.08	3.22	2.93	3.07	3.29
**COR**	**xCoP**	0.33	0.28	0.25	0.35	0.30	0.30	0.29	0.32	0.32	0.23
	**yCoP**	0.05	−0.03	0.04	0.02	0.02	0.01	0.04	−0.06	0.01	0.03
	**Fz**	0.91	0.90	0.9	0.91	0.90	0.90	0.89	0.91	0.90	0.89

**Table 18:** Mean results for three-dataset-trained NN in random exercise.

	Model	1	2	3	4	5	6	7	8	9	10
**NRMSE**	**xCoP**	7.52	10.7	9.44	8.71	8.11	8.29	7.87	9.99	7.89	9.18
	**yCoP**	15.0	19.0	19.7	15.8	13.4	12.9	14.9	13.4	12.6	15.1
	**Fz**	4.25	6.25	6.89	5.51	4.30	4.08	4.63	4.23	3.37	4.10
**COR**	**xCoP**	0.80	0.73	0.73	0.77	0.78	0.77	0.77	0.75	0.79	0.72
	**yCoP**	0.46	0.38	0.40	0.37	0.40	0.38	0.33	0.42	0.39	0.40
	**Fz**	0.98	0.93	0.93	0.96	0.98	0.98	0.96	0.98	0.99	0.98

**Table 19:** Comparison of the analytical and NN NRMSE results.

		xCoP Left	yCoP Left	Fz Left	xCoP Right	yCoP Right	Fz Right
**STC**	**An.**	1.708	5.968	1.103	1.690	4.330	1.219
	**NN**	1.934	7.089	1.083	1.920	4.849	1.142
**AP**	**An.**	2.520	10.63	1.504	2.203	11.28	1.505
	**NN**	2.540	6.249	1.388	2.289	7.398	1.409
**ML**	**An.**	6.512	12.85	2.758	7.114	13.16	2.867
	**NN**	4.083	7.697	1.685	4.942	6.941	1.712

**Table 20:** Comparison of the analytical and NN correlation results.

		xCoP Left	yCoP Left	Fz Left	xCoP Right	yCoP Right	Fz Right
**STC**	**An.**	0.924	0.170	0.982	0.900	−0.080	0.984
	**NN**	0.870	0.162	0.849	0.870	0.000	0.849
**AP**	**An.**	0.996	−0.121	0.919	0.997	0.181	0.924
	**NN**	0.996	0.663	0.910	0.996	0.669	0.919
**ML**	**An.**	0.590	0.364	1.000	0.380	0.555	1.000
	**NN**	0.831	0.508	0.999	0.759	0.362	0.999

**Figure A1:**
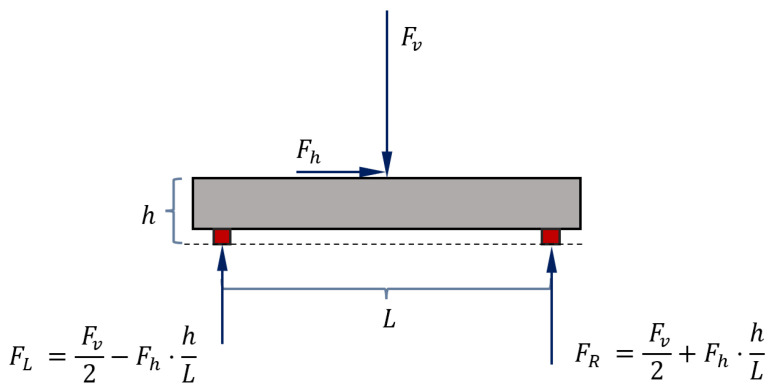
Forces diagram.

## Data Availability

Data are not available due to privacy restrictions.

## Abbreviations

The following abbreviations are used in this manuscript:

GRF	Ground Reaction Force.
CoP	Center of Pressure.
NN	Neural Network.
STC	Static Exercise.
AP	Antero-Posterior Exercise.
ML	Medio-Lateral Exercise.
ROT	Rotation Exercise.
RND	Random Exercise.
RMSE	Root Mean Squared Error.
NRMSE	Normalized Root Mean Squared Error.
COR	Pearson Correlation Coefficient.

## Appendix A

To make the calculation as realistic as possible, the following assumptions were used in the calculations (see Figure A1):

- The vertical offset between the top of the plate and the point where the sensor makes the measurement is h = 22 mm, where L = 200 mm is the horizontal distance between the force sensors.
- The horizontal component of the force is significantly influenced by the angle at which the legs are positioned. According to the values given in Table 2 of reference [7], an intermediate angle between the neutral posture and legs in a wide-stance position was used: $F_{v}\;=\;373\;N$ and $F_{h}\;=\;57/2\;=\;28.5\;N$.
- In order to estimate the error caused by horizontal forces, we consider a force applied at a known point, specifically the center of the plate.
- Only the error generated by the upper force plate is calculated, as its h/L ratio is higher. In addition, for the lower platform where global CoP is measured, the error is compensated when the load of both feet is applied (horizontal forces have opposite directions).

Now, we calculate a new CoP location from those reactions values, but neglecting h, as if they were applied in the same plane as the force. This emulates the calculation that we are currently performing in our device, as in [27], given by the moment generated from the difference in vertical reaction forces ($F_{R}$ and $F_{L}$). In this way, the new position of the CoP is not exactly at the center of the plate, and the existing deviation represents the error produced by the existence of the horizontal component $F_{h}$ and the vertical offset h.

(A1) $$F_{L}=186.50-3.135=183.365\;N$$

(A2) $$F_{R}=186.50+3.135=189.635\;N$$

(A3) $$M_{x}=(F_{R}-F_{L})\cdot L/2=627\;N\cdot mm$$

(A4) $$\mathrm{yCoP}=M_{x}/F_{v}=1.68\;mm$$

The Equation (A4) represents the displacement of the yCoP from the center, which corresponds to the measurement error. This is less than the error made by the prototype when applying purely vertical forces under controlled conditions (2.7 mm), so it can be considered negligible.
